# Supplementary figures and images for: Nonsmoking after simultaneous alcohol abstinence and smoking cessation program was associated with better drinking status outcome in Japanese alcohol-dependent men: A prospective follow-up study
Source: PLoS One. 2023 Mar 29;18(3):e0282992. doi: 10.1371/journal.pone.0282992 (PMC10057780; doi:10.1371/journal.pone.0282992)

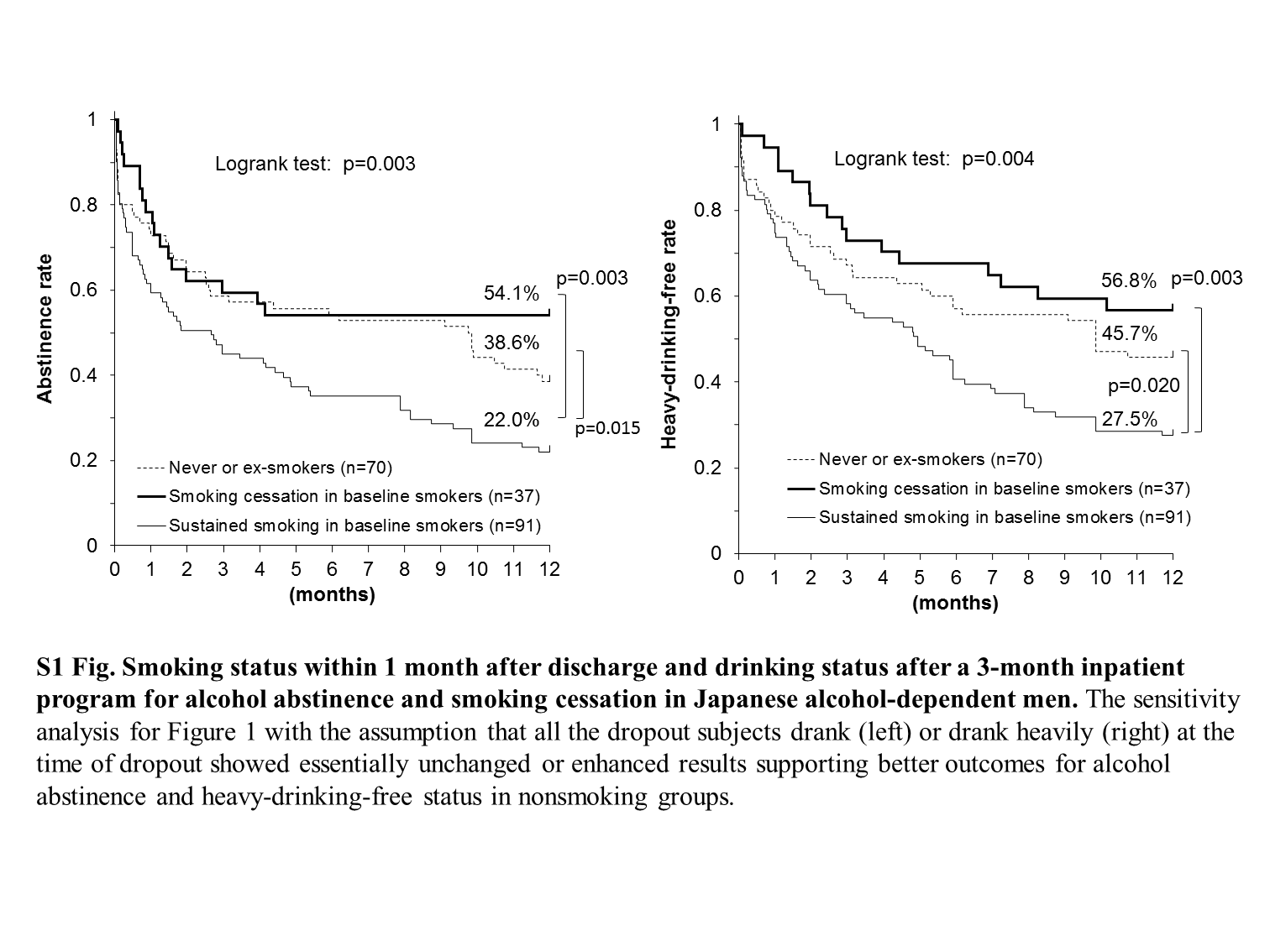

Supplement: S1 Fig — The sensitivity analysis for Fig 1 with the assumption that all the dropout subjects drank (left) or drank heavily (right) at the time of dropout showed essentially unchanged or enhanced results supporting better outcomes for alcohol abstinence and heavy-drinking-free status in nonsmoking groups. (TIF) [file pone.0282992.s002.tif]

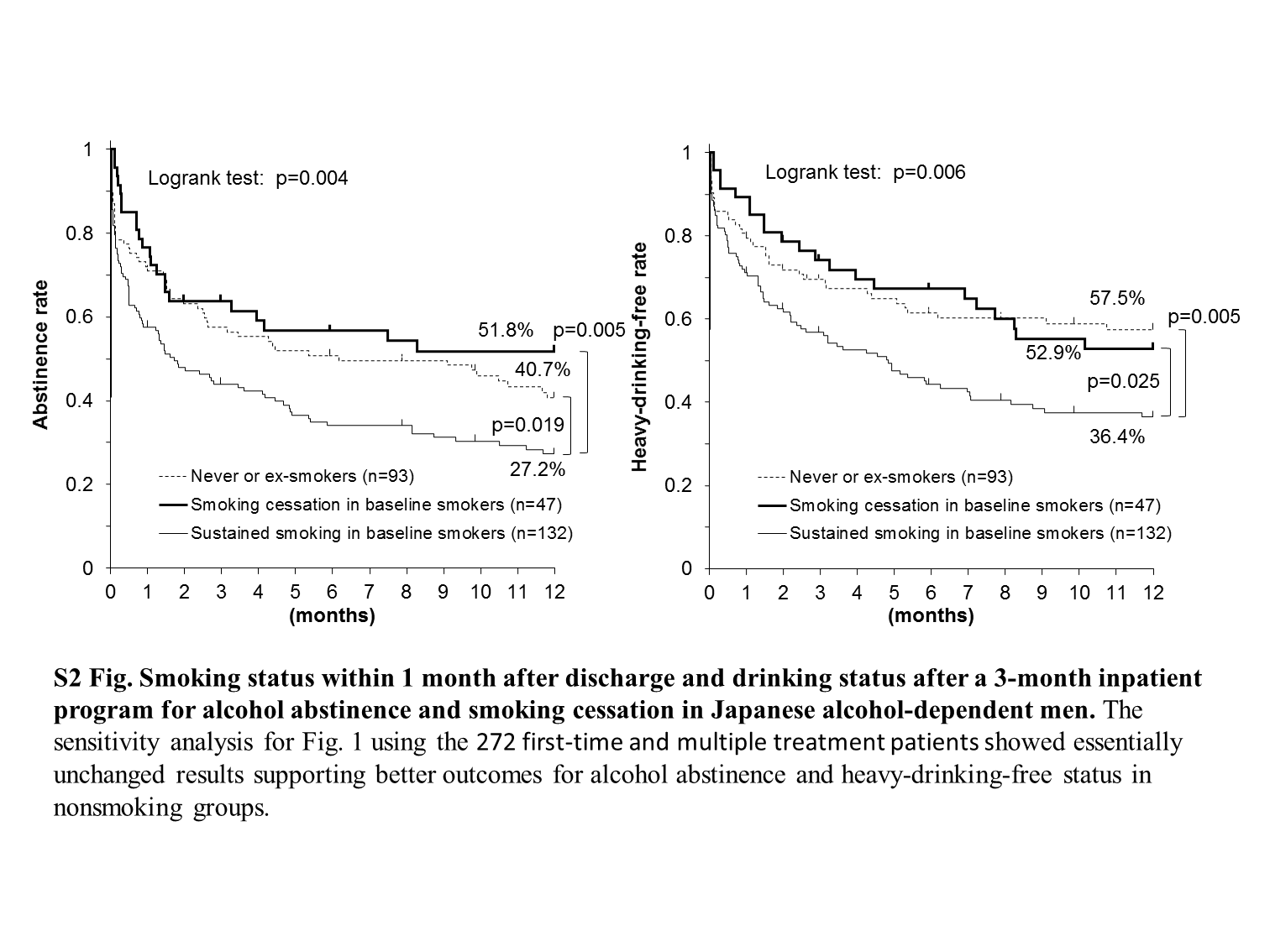

Supplement: S2 Fig — The sensitivity analysis for Fig 1 using the 272 first-time and multiple treatment patients showed essentially unchanged results supporting better outcomes for alcohol abstinence and heavy-drinking-free status in nonsmoking groups. (TIF) [file pone.0282992.s003.tif]

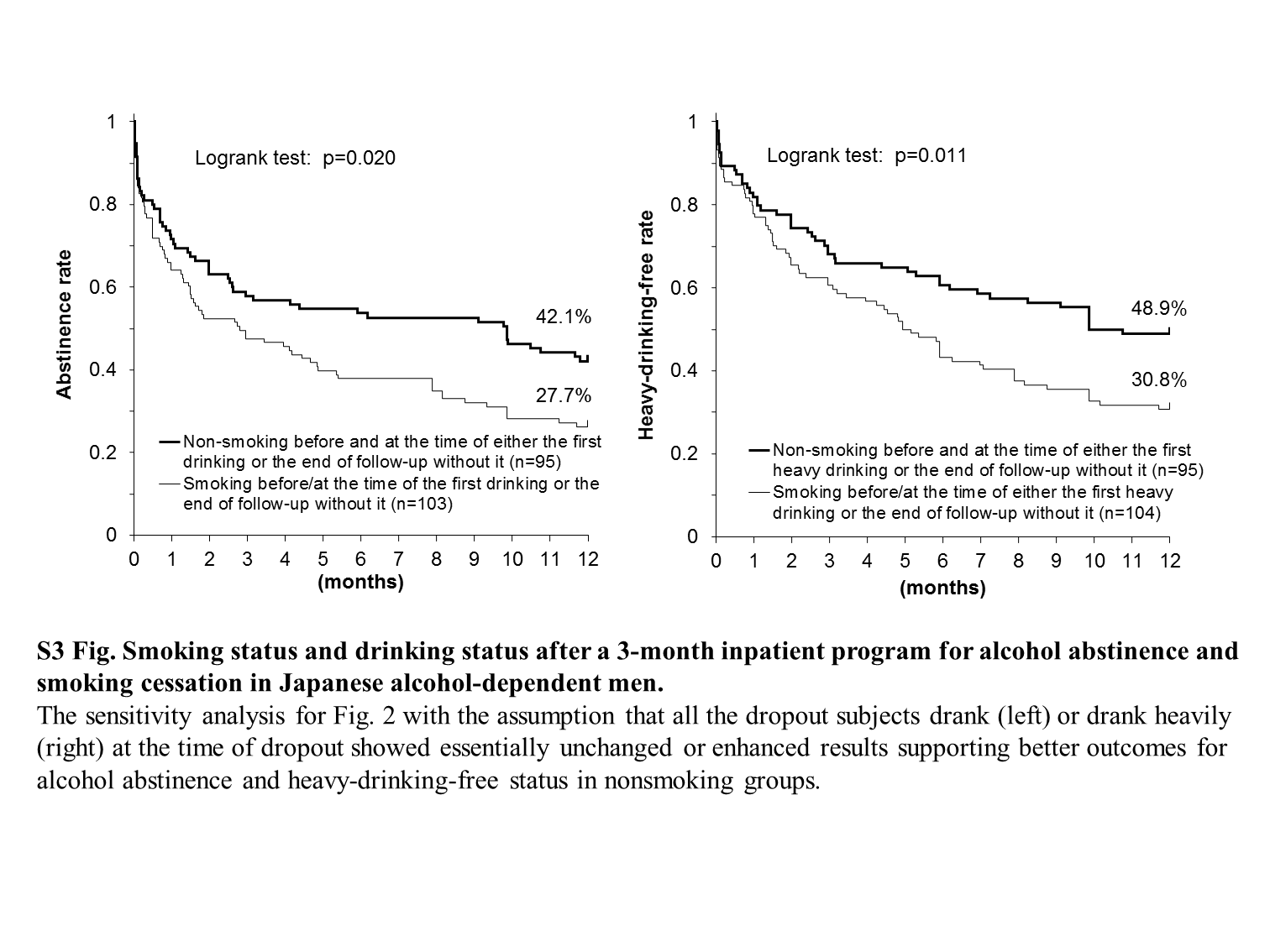

Supplement: S3 Fig — The sensitivity analysis for Fig 2 with the assumption that all the dropout subjects drank (left) or drank heavily (right) at the time of dropout showed essentially unchanged or enhanced results supporting better outcomes for alcohol abstinence and heavy-drinking-free status in nonsmoking groups. (TIF) [file pone.0282992.s004.tif]

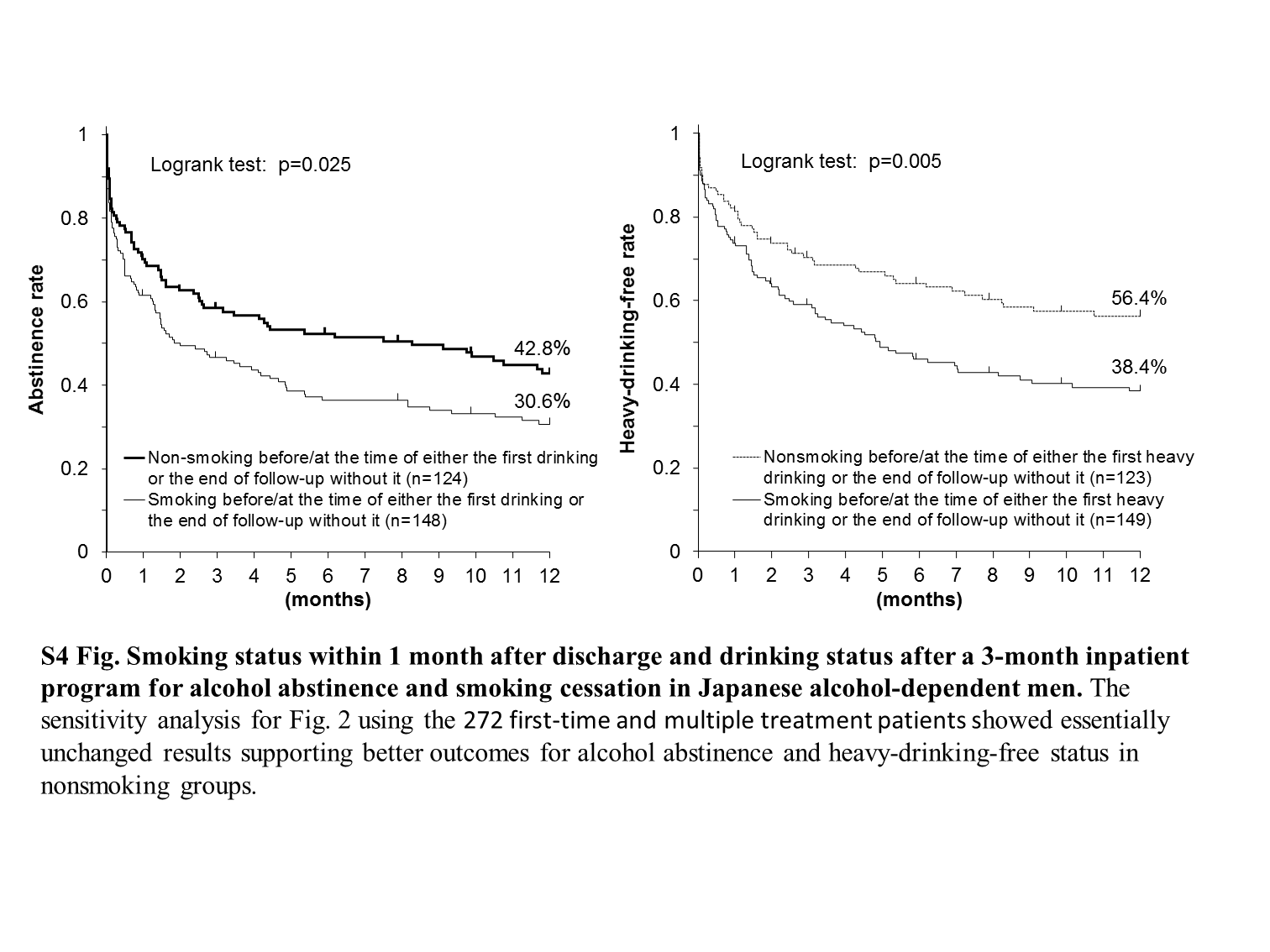

Supplement: S4 Fig — The sensitivity analysis for Fig 2 using the 272 first-time and multiple treatment patients showed essentially unchanged results supporting better outcomes for alcohol abstinence and heavy-drinking-free status in nonsmoking groups. (TIF) [file pone.0282992.s005.tif]
